# Downregulation of Heat Shock Protein 70 Impairs Osteogenic and Chondrogenic Differentiation in Human Mesenchymal Stem Cells

**DOI:** 10.1038/s41598-017-18541-1

**Published:** 2018-01-11

**Authors:** Chenghai Li, Kristifor Sunderic, Steven B. Nicoll, Sihong Wang

**Affiliations:** Department of Biomedical Engineering, City University of New York-City College, 160 Convent Avenue, New York, NY 10031 USA

## Abstract

Human mesenchymal stem cells (hMSCs) show promise for bone and cartilage regeneration. Our previous studies demonstrated that hMSCs with periodic mild heating had enhanced osteogenic and chondrogenic differentiation with significantly upregulated heat shock protein 70 (HSP70). However, the role of HSP70 in adult tissue regeneration is not well studied. Here, we revealed an essential regulatory mechanism of HSP70 in osteogenesis and chondrogenesis using adult hMSCs stably transfected with specific shRNAs to knockdown HSP70. Periodic heating at 39 °C was applied to hMSCs for up to 26 days. HSP70 knockdown resulted in significant reductions of alkaline phosphatase activity, calcium deposition, and gene expression of Runx2 and Osterix during osteogenesis. In addition, knockdown of HSP70 led to significant decreases of collagens II and X during chondrogenesis. Thus, downregulation of HSP70 impaired hMSC osteogenic and chondrogenic differentiation as well as the enhancement of these processes by thermal treatment. Taken together, these findings suggest a putative mechanism of thermal-enhanced bone and cartilage formation and underscore the importance of HSP70 in adult bone and cartilage differentiation.

## Introduction

Bone marrow derived human mesenchymal stem cells (hMSCs) have shown great potential for tissue-engineering applications. This is due to their ability to differentiate into different tissue types such as bone, cartilage, adipose tissue, and muscle^1^. Our own previous studies revealed that periodic heat shock at 41 °C enhanced osteogenic^2^ and chondrogenic differentiation of hMSCs^3^. We also observed that heat shock protein 70 (HSP70), a highly conserved protein family member in mammalian cells, was significantly upregulated by heat shock in differentiated hMSCs^2,3^, indicating a potential role of HSP70 in hMSC bone and cartilage differentiation. HSP70 plays an important role during development and repair under normal cellular homeostasis and stress conditions via its molecular chaperone functions^4,5^. Most of the heat shock proteins are constitutively expressed in animals and humans. The stress-inducible HSP70 is rapidly synthesized in response to many physiological (e.g. hyperthermia, energy depletion, inflammation, exercise, and differentiation) and pathological (e.g. hypoxia, ischemia, acidosis, viral infection, and reactive oxygen species) conditions^6,7^.

The cellular protection function of HSP70 induced by mild heating provides thermo-tolerance to cells in culture and animals from extreme heat-induced cellular damage^8,9^. In the case of tissue injury, HSP70 serves as a modulating molecule for immune and inflammatory responses. For example, macrophages with accumulated HSP70 had decreased secretion of the inflammatory cytokines, TNF-α and IL-1^10^, and HSP70 enabled cells to have a high tolerance to inflammatory cytokines. Accumulating evidence also indicates that HSP70 plays specific roles in cell differentiation and tissue development. One study on erythropoiesis showed that HSP70 bound to apoptosis-inducing factor (AIF) to block AIF-induced apoptosis, thus enabling the differentiation of erythroblasts^11^. Another study showed an increase of HSP70 protein expression in the cerebral hemispheres and kidney throughout the postnatal development of rats^12^. A study of endochondral bone development in mouse embryos correlated HSP expressions to different stages of bone and cartilage differentiation^13^. Another study linked HSP70 to differentiation of mammalian osteoblasts^14^. However, the role of HSP70 in bone and cartilage tissue engineering using adult stem cells, especially during heat-enhanced bone formation, has not been explored.

Considering non-specificity of chemical inhibitors, target-specific HSP70 shRNAs were used to knock down HSP70 expression in hMSCs, and the role of HSP70 in osteogenic and chondrogenic differentiation of hMSCs was examined with and without HSP70 knockdown. An early study indicated that heating 1.5–3 °C above regular body temperature plays a role in bone growth stimulation in rats and dogs^15^. Further, regular mild exercise has been shown to increase body core temperature to an average temperature of 39 °C^16–18^, inhibit bone and cartilage degradation and improve knee function in parallel with structural improvement in patients with knee osteoarthritis^19–21^. Therefore, in this study hMSCs were exposed to mild heating at 39 °C for one hour once every other day during osteogenic and chondrogenic differentiation of 3 to 4 weeks. Conventional tissue culture plates (Young’s modulus of 3.5 GPa)^2^ were chosen in our osteogenic studies due to their similar stiffness to bone. Osteogenic differentiation of hMSCs is typically assessed by monitoring alkaline phosphatase (ALP) activity, an early marker of osteogenic differentiation, and calcium deposition, a late indicator^22,23^. Pellet cultures were performed for chondrogenic differentiation of hMSCs. Immunohistochemistry for collagen type II, type X, and aggrecan was performed on chondrogenic pellets. It was found that HSP70 knockdown impaired osteogenic and chondrogenic differentiation and diminished heat-enhanced osteogenesis and chondrogenesis in hMSCs. Overall, these data demonstrate that HSP70 plays a direct and key role in heat-enhanced hMSC osteogenesis and chondrogenesis. Moreover, this study may result in a potential new approach toward prevention and treatment of diseases related to bone and cartilage loss.

## Results

### Periodic exposure of hMSCs to mild heat shock at 39 °C upregulated HSP70 expression, enhanced osteogenic and chondrogenic differentiation

**Figure 1:**
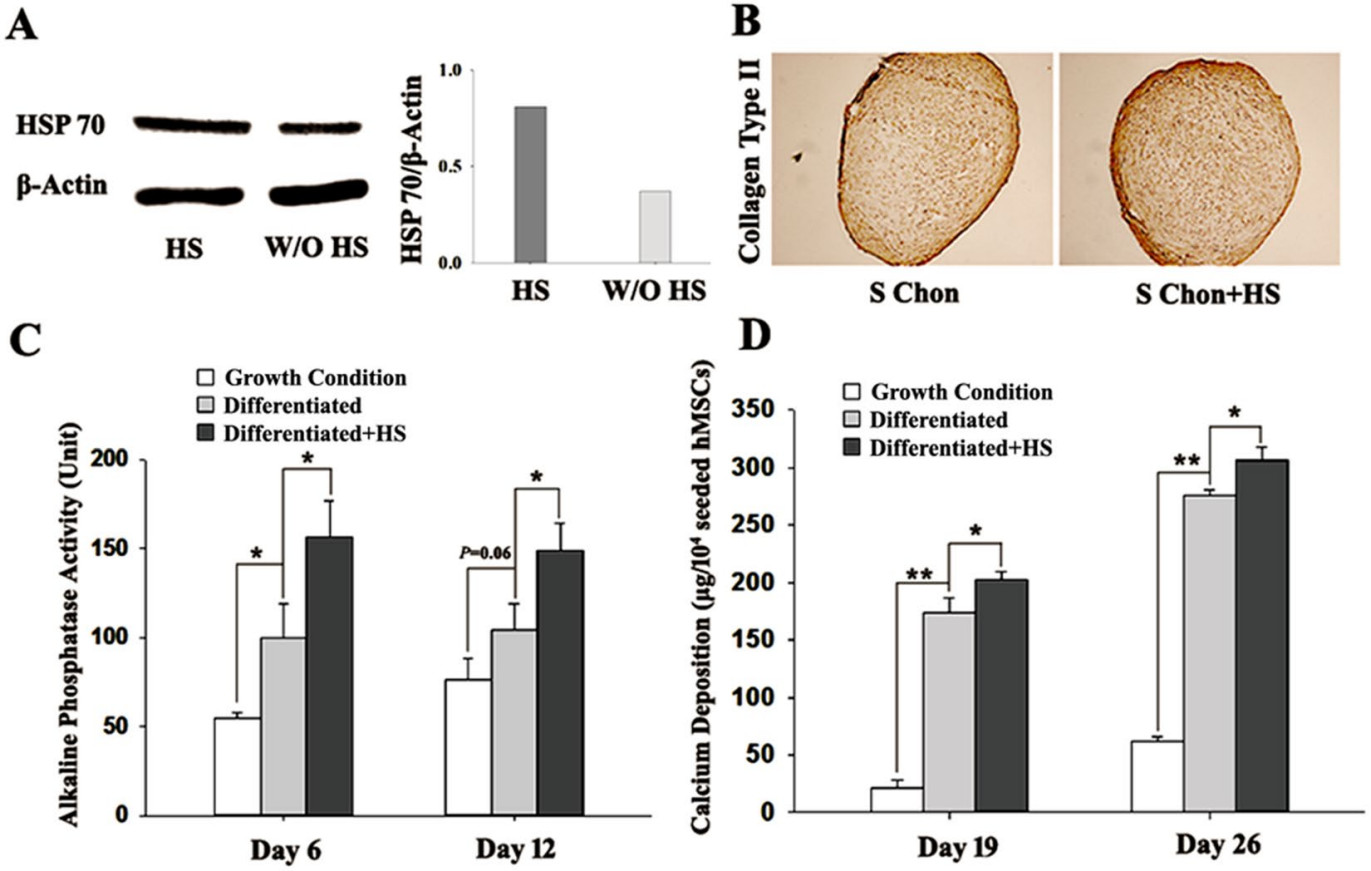
Expression of HSP70, osteogenic and chondrogenic markers in hMSCs transfected with a scrambled shRNA and treated with mild heat shock at 39°C for one hour once every other day. (**A**) Western Blot (WB) analysis of HSP70 in growth medium from a full WB membrane in Figure S2 and density quantification of bands, respectively, (**B**) Immunohistochemical staining of collagen II in 3D pellets at Day 17 during chondrogenesis, (**C**) ALP activity at Day 6 and 12, and (**D**) calcium deposition at Day 19 and 26 during osteogenesis. One enzyme Unit of ALP is the amount of enzyme which releases 1 nmol p-nitrophenol per 15 minutes. Each sample had 10^4^ seeded hMSCs and N = 4. HS: heat shock; S: scrambled; Chon: chondrogenic differentiation. *P < 0.05; **P < 0.01.

As shown in Fig. 1A, mild heat shock at 39 °C for one hour followed by 16 hours incubation at 37 °C increased the inducible HSP70 expression in hMSCs with growth medium. Effects of periodic mild heating at 39 °C for one hour once every two days on hMSC chondrogenic differentiation at Day 17 are illustrated in Fig. 1B with upregulated type II collagen in pellet cultures using hMSCs transfected with scrambled shRNA. For osteogenic differentiation of hMSCs transfected with scrambled shRNA, Fig. 1C and D shows characteristic osteogenesis indicated by upregulation of ALP activity and calcium deposition in cultures with differentiation medium. Furthermore, the same heating pattern also significantly enhanced ALP activity at Day 6 (Fig. 1C) and calcium deposition (Fig. 1D) in differentiated hMSCs with the scrambled shRNA. These differentiation data are consistent with our previous reports^2,3^, indicating that osteogenic and chondrogenic functions of hMSCs as well as heat-enhancing effects on both types of differentiation were not altered by non-specific shRNA transfection.

### HSP70 shRNA specifically knocked down HSP70 expression in hMSCs and confirmation of hMSC surface markers after HSP70 knockdown

**Figure 2:**
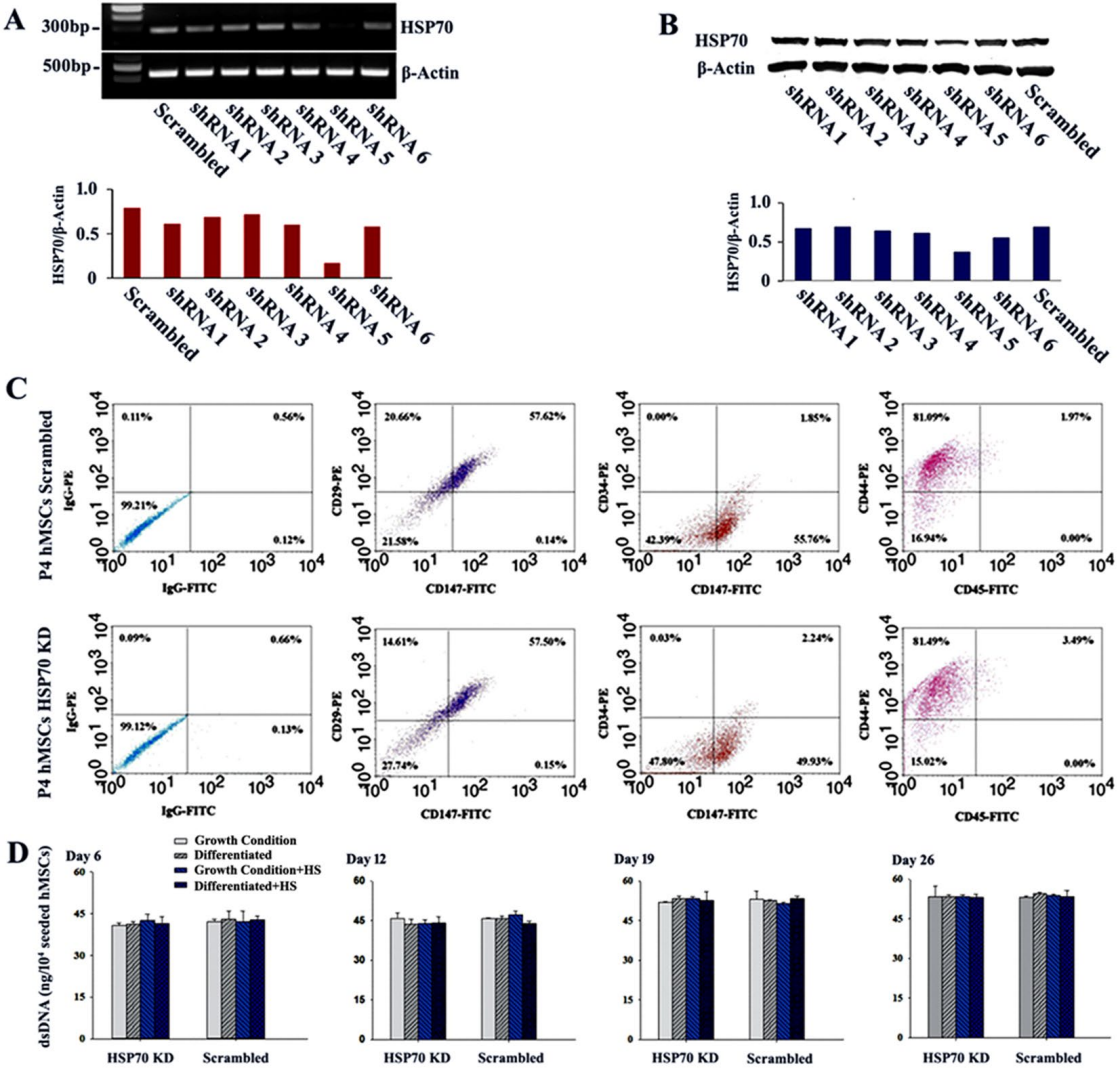
HSP70 knockdown efficiency, surface marker expression, and proliferation of hMSCs. (**A**) RT-PCR results from full DNA gel images in Figure S3 and (**B**) Western Blot (WB) results from a full WB membrane in Figure S4 and band density quantification of heated hMSCs transfected with a scrambled shRNA (control) and HSP70 shRNAs #1 to #6; (**C**) Representative dot plots of flow cytometric analysis of surface marker expressions of hMSCs transfected with scrambled shRNA (top row) and HSP70 shRNA#5 (bottom row). Fluorescence thresholds were determined by fluorescence intensities of hMSCs with negative control antibodies IgG-FITC and IgG-PE; (**D**) dsDNA contents of hMSCs during osteogenic differentiation at Day 6, 12, 19, and 26. N = 4. KD: knockdown; HS: heat shock.

To investigate the role of HSP70 in hMSC osteogenic and chondrogenic differentiation, an shRNA-based gene silencing strategy was employed to knockdown HSP70 expression in hMSCs. The efficiency of HSP70 knock down using shRNAs was confirmed in heated hMSCs at both mRNA and protein levels (Fig. 2A and B). RT-PCR showed that one out of six shRNAs (shRNA5 targeting HSPA1B) dramatically downregulated HSP70 expression in hMSCs (Fig. 2A). Densitometry analysis confirmed that the expression of HSP70 mRNA was reduced by more than 70% in heated hMSCs transfected with shRNA5 compared with those transfected with a scrambled shRNA. Consistent with the reduction of mRNA level in heated hMSCs, HSP70 protein expression in these samples decreased to 50% of the control treated with scrambled shRNA (Fig. 2B). In this study, passage 3 hMSCs were used for HSP70 gene silencing or the control cultures with shRNA5 or scrambled shRNA, respectively, and passage 4 hMSCs were used for osteogenic and chondrogenic differentiation.

To examine whether HSP70 knockdown changed the surface marker expression of hMSCs, we performed flow cytometric analysis using hMSCs at passage 4. In Fig. 2C and Table 1, flow cytometric analysis shows that isolated hMSCs of passage 4 were positive for surface markers CD29, CD44, and CD147, and negative for CD34 and CD45. Furthermore, there was no significant difference in the expression of these surface markers between hMSCs with HSP70 knockdown and the control (Table 1). Figure 2C includes representative flow cytometry profiles of CD29, CD34, CD44, CD45, and CD147 in passage 4 hMSCs with (bottom row in Fig. 2C) or without (top row in Fig. 2C) HSP70 knockdown.

**Table 1:** Percentage of surface marker expression of CD29, CD34, CD44, CD45, and CD147 in hMSCs with HSP70 knockdown or scrambled shRNA analyzed by flow cytometry.

Human MSCs	CD29 (%)	CD34 (%)	CD44 (%)	CD45 (%)	CD147 (%)
Scrambled	77.41 ± 1.78	2.43 ± 1.47	81.15 ± 2.19	2.17 ± 0.56	54.89 ± 2.36
HSP70 Knockdown	71.79 ± 2.31	2.44 ± 0.40	84.14 ± 0.37	2.53 ± 0.08	56.27 ± 0.30

The proliferation of hMSCs after HSP70 knockdown was also studied. During hMSC osteogenic differentiation, cells were harvested on Day 6, 12, 19, and 26 and samples were analyzed for dsDNA content. As shown in Fig. 2D, there was a slow increase in dsDNA during the early differentiation stage, which plateaued on Day 19. Figure 2D also shows that there was no significant difference in dsDNA content between samples in growth and osteogenic conditions on the same days. In addition, hMSCs with HSP70 knockdown had a similar dsDNA content during osteogenic differentiation as the control samples with scrambled shRNA. Furthermore, mild heating at 39 °C for one hour once every other day did not affect dsDNA content in samples of different culture conditions on the same culture days (Fig. 2D). Thus, HSP70 knockdown did not affect the MSC proliferation.

These data of hMSCs stably transfected with HSP70 shRNA demonstrate that knockdown of HSP70 did not change proliferation of hMSCs or their stemness in terms of the MSC surface marker expressions.

### Expression of HSP70 in hMSCs with HSP70 knockdown during osteogenesis and chondrogenesis

**Figure 3:**
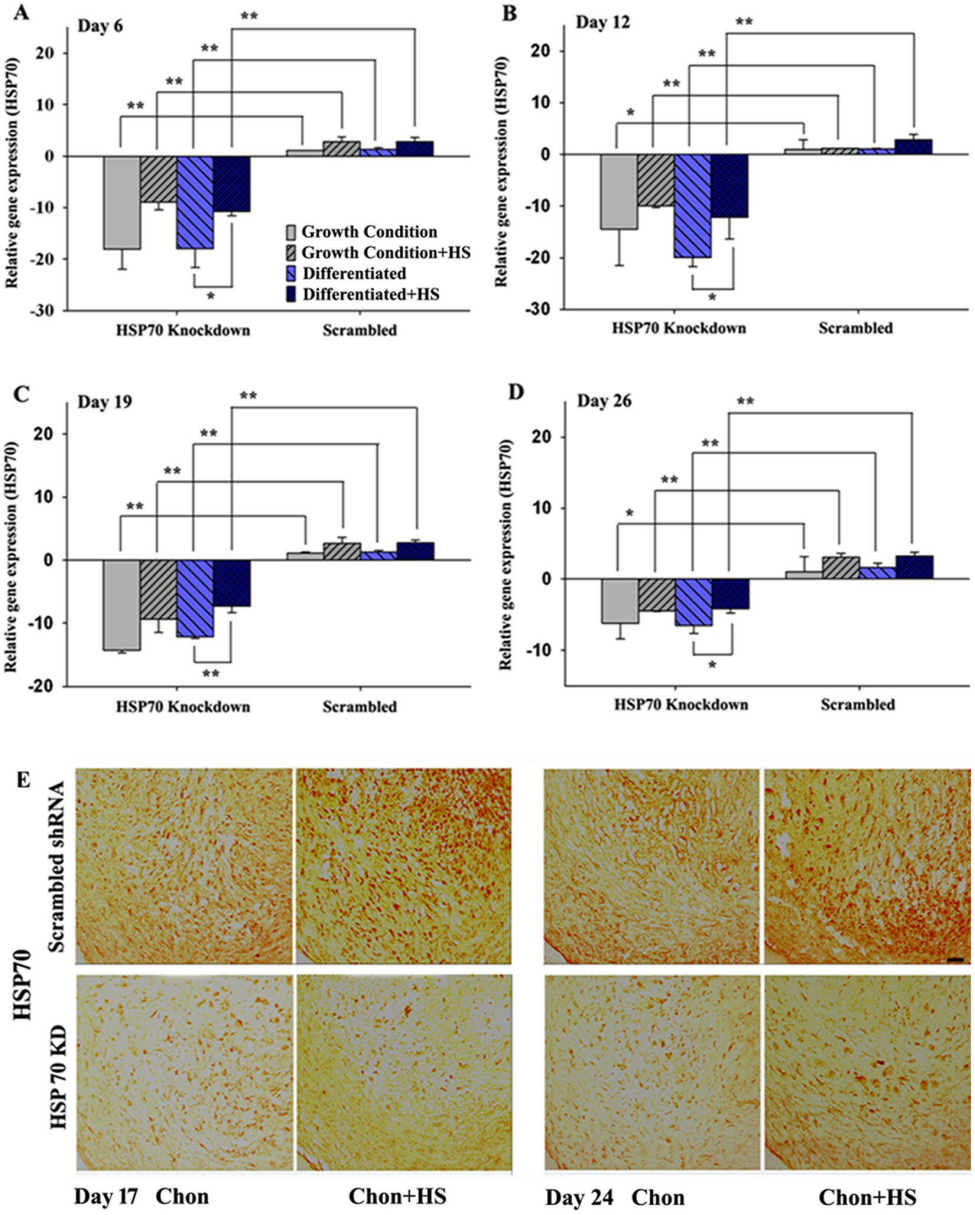
HSP70 expression in hMSCs with HSP70 knockdown during osteogenesis and chondrogenesis. HSP70 expression measured by Real-time RT-PCR in hMSCs during growth and osteogenic differentiation with and without heat shock at Day 6 (**A**), 12 (**B**), 19 (**C**), and 26 (**D**). Representative images of immunohistochemical staining of HSP70 in pellet culture samples at day 17 and 19 during chondrogenesis (**E**); Scale bar: 50 $\mu$m (Chon: chondrogenic differentiation; HS: heat shock; KD: knockdown). *P < 0.05; **P < 0.01. N = 4.

To confirm HSP70 knockdown efficiency in differentiated hMSCs during osteogenesis, HSP70 mRNA expression levels were assessed by real time RT-PCR. In Fig. 3A–D, real time RT-PCR analysis on Day 6, 12, 19, and 26 of osteogenic differentiation showed a significant reduction of HSP70 expression in hMSCs stably transfected with HSP70 shRNA5 compared with controls transfected with scrambled shRNA. Interestingly, periodic mild heat shock did induce slightly more HSP70 expression in heated than non-heated hMSCs with HSP70 knockdown at all four time points during osteogenic differentiation (Fig. 3A–D). This may be explained by an incomplete knockdown of HSP70 in hMSCs by shRNA5.

As shown in Fig. 3E, pellets with the scrambled shRNA had increased staining of HSP70 in heated compared to non-heated conditions on day 17 and 24 during chondrogenesis. Meanwhile, Fig. 3E pellets with HSP70 knockdown (“HSP70 KD”) had much lower HSP70 expression compared to controls with scrambled shRNA on both days, and mild heat shock slightly rescued the dramatic reduction of HSP70 expression in the knockdown samples. However, the slightly rescued HSP70 expression could not reach the same high level of HSP70 expression after heat shock in the control pellets with scrambled shRNA. Taken together, these results confirm that HSP70 was efficiently knocked down in hMSCs undergoing chondrogenesis in pellet culture, and that periodic mild heat shock slightly rescued HSP70 expression in HSP70 knockdown pellets likely due to the fact that shRNA5 did not completely abrogate HSP70 expression.

### HSP70 knockdown significantly impaired hMSC osteogenesis

**Figure 4:**
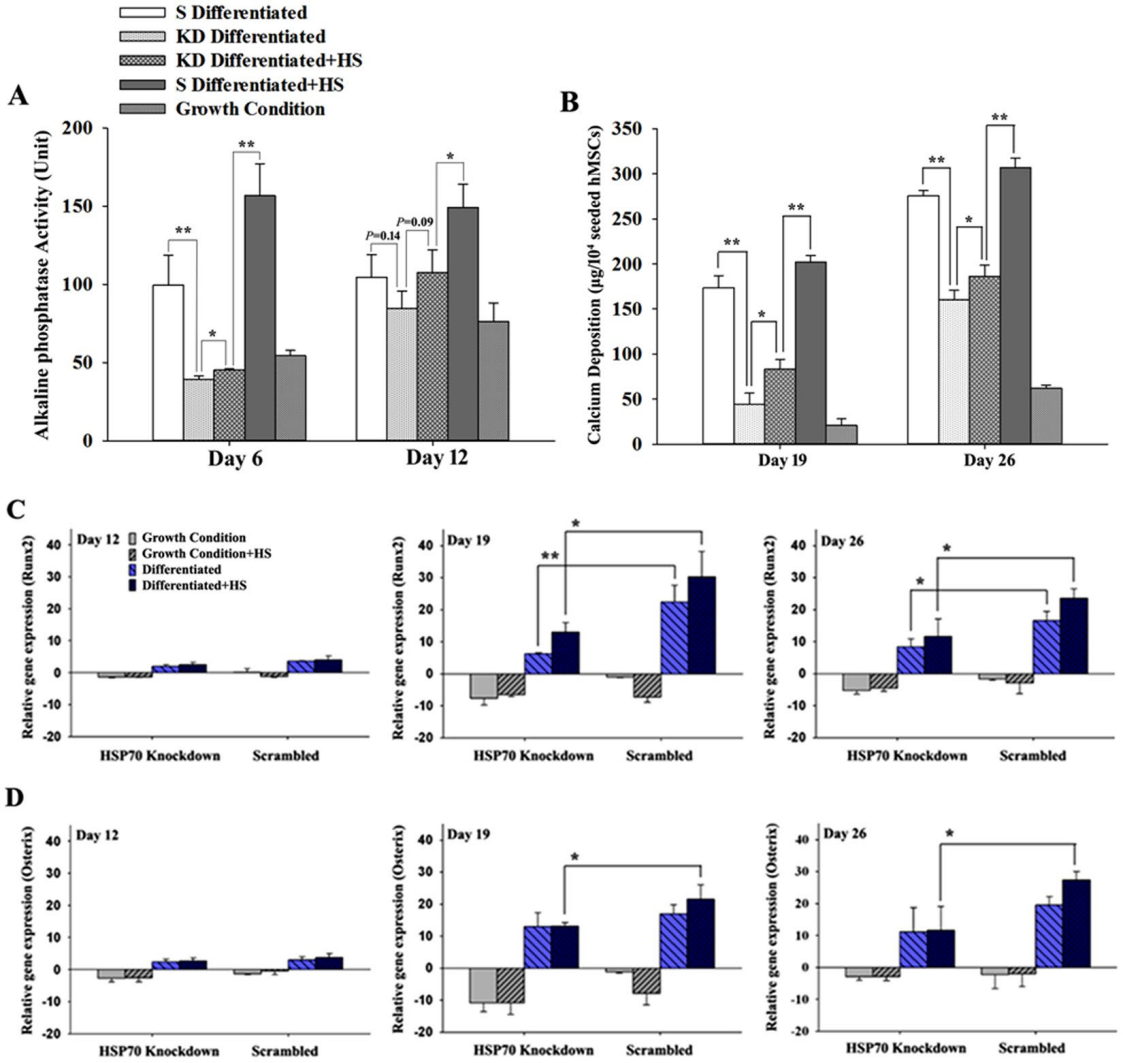
Comparison between samples of HSP70 knockdown and controls in terms of ALP activity (**A**), calcium deposition (**B**) and real time PCR data of gene expression of Runx2 (**C**) and Osterix (**D**) during osteogenic differentiation in hMSCs. One enzyme unit of ALP is the amount of enzyme which releases 1 nmol p-nitrophenol per 15 minutes. Each sample had 10^4^ seeded hMSCs. N = 4. S: scrambled; KD: HSP70 knockdown; HS: heat shock. *P < 0.05; **P < 0.01.

Osteogenic differentiation of hMSCs was monitored by ALP activity and calcium deposition. Though there were some degrees of upregulation of ALP at Day 12 (Fig. 4A) and calcium deposition at Day 19 and Day 26 (Fig. 4B) in differentiated samples with HSP70 knockdown, statistically significant reductions of these two differentiation markers were observed after HSP70 knockdown (p < 0.01). In detail, ALP activity was diminished to 45 ± S.D. units from 157 ± S.D. units at Day 6 in heated samples with HSP70 knockdown (Fig. 4A), a 71% reduction. Compared to the control, knockdown of HSP70 resulted in a reduction of calcium deposition by 75% in non-heated hMSCs and 59% in heated hMSCs at Day 19 (Fig. 4B). Additionally, knockdown of HSP70 resulted in a 41.84% reduction in non-heated hMSCs and 39.18% in heated hMSCs (Fig. 4B) at Day 26 during differentiation.

Periodic mild heating slightly recovered the diminished ALP activity at Day 6 and calcium deposition at Days 19 and 26 in hMSCs with HSP70 knockdown as shown in Fig. 4A and B (p < 0.05). However, the slight recovery of ALP and calcium from heating in HSP70 knockdown samples could not compensate for the expression reduction of osteogenic markers due to HSP70 knockdown. For example, 45 ± S.D. units of ALP from the heated HSP70 knockdown samples is much lower than 99 ± S.D. units of ALP from the non-heated samples with scrambled shRNA (Fig. 4A Day 6). Similarly, there was significantly less calcium (83.11 μg) in heated HSP70 knockdown samples than non-heated controls (173.78 μg) (Fig. 4B Day 19). The complete results of ALP activities and calcium deposition for growth and osteogenic conditions with and without HSP70 knockdown are included in Tables S1 and S2 in supplementary materials.

We further examined the expression of osteogenesis-specific genes, Runx2 and Osterix during MSC osteogenesis. Figure 4C and D include results of Runx2 and Osterix gene expression assessed by real-time RT-PCR on Day 12, 19, and 26 during osteogenic differentiation. As expected and shown in Fig. 4C and D, undifferentiated hMSCs showed very low expression levels of Runx2 and Osterix compared with the differentiated hMSCs, while both Runx2 and Osterix expression gradually increased in osteogenic samples from early to late stage differentiation. Day 12 data in Fig. 3C and D show that there was no significant difference in the expression of Runx2 and Osterix between hMSCs with HSP70 knockdown and control hMSCs (i.e. hMSCs with the scrambled shRNA). However, HSP70 knockdown significantly reduced the Runx2 expression on Days 19 and 26 (Fig. 4C) during osteogenesis regardless of heating. Similarly, inhibition of HSP70 significantly decreased the Osterix expression in heated hMSCs on Day 19 and 26 (Fig. 4D) during osteogenic differentiation.

### Downregulation of chondrogenic markers in hMSC pellet culture after HSP70 knockdown

**Figure 5:**
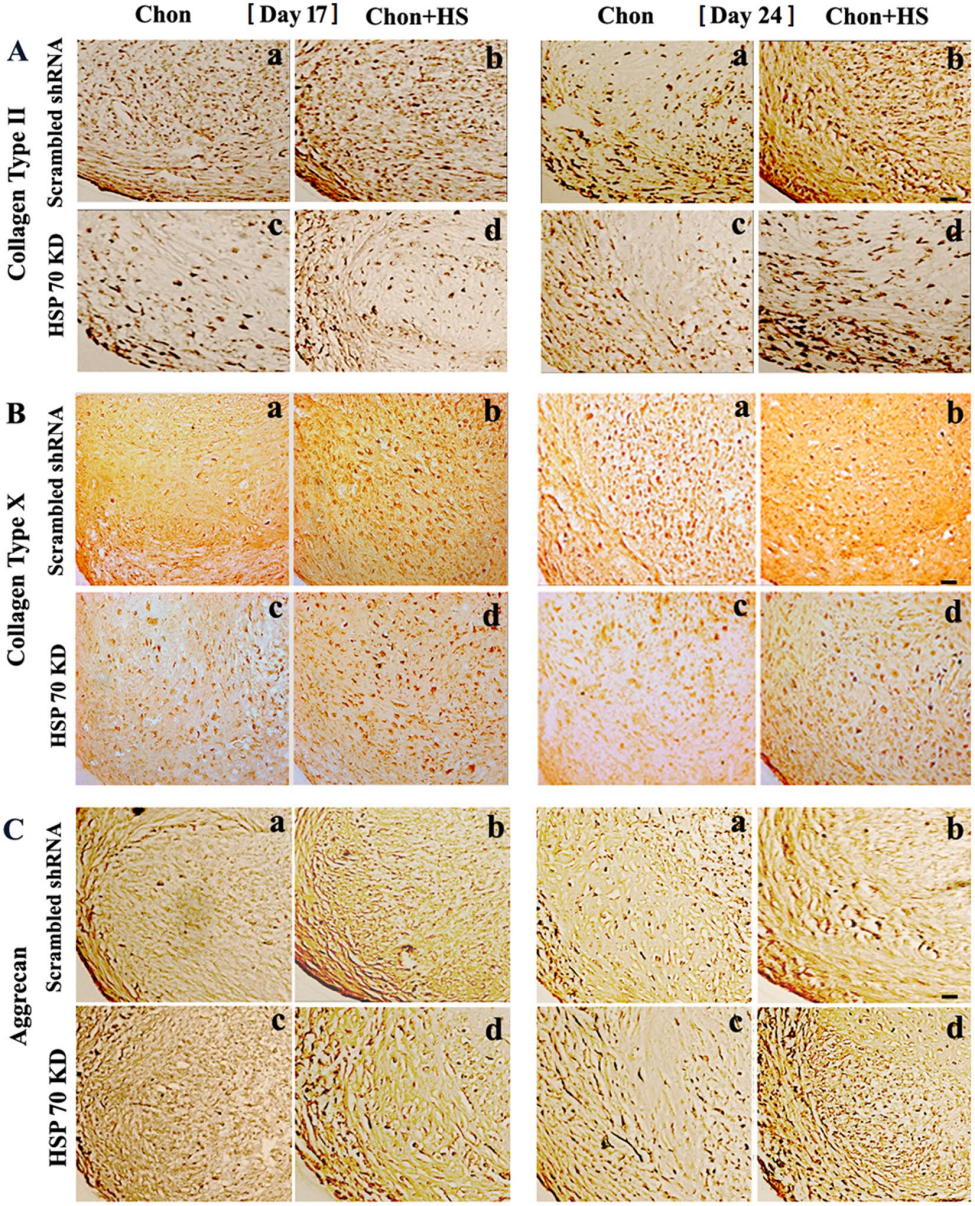
Representative images of immunohistochemical staining of collagen type II (**A**), collagen type X (**B**), and aggrecan (**C**) in pellet culture samples during chondrogenesis. Scale bar: 50 ${\mu }$m. (Chon: chondrogenic differentiation; HS: heat shock; KD: knockdown).

3D pellet culture was performed for chondrogenic differentiation of hMSCs with and without HSP70 knockdown. Pellets were exposed to mild heat shock at 39 °C for one hour every other day. The pellet samples were examined for collagen II, collagen X, and aggrecan expression by immunohistochemistry at day 17 and 24 during chondrogenesis (Fig. 5). Table 2 includes quantified results of immunohistochemical staining intensity of collagen II, X and aggrecan. The mild heating at 39 °C upregulated the expression of chondrogenic markers, collagen II and aggrecan, and the hypertrophic cartilage marker, collagen X, in chondrogenic pellets with scrambled shRNA at days 17 and 24 (Fig. 5A,B and C, comparisons between panels a and b). HSP70 knockdown reduced collagen II and X expressions dramatically in chondrogenic pellets (Fig. 5A and B, comparisons between panels a and c, b and d; Table 2). Similar to osteogenic conditions, there was a slight increase in collagen II and X expression in heated HSP70 knockdown pellets that might be caused by partial knockdown of HSP70 (Fig. 5A and B, comparisons between panels c and d). Interestingly, HSP70 knockdown did not result in dramatic decrease of aggrecan expression in non-heated and heated conditions (Fig. 5C comparisons between panels a and c, b and d). Additionally, heating itself could lead to the significant increase of aggrecan expression though the samples have the same 70% HSP70 knockdown (Fig. 5C, comparisons between panels c and d).

**Table 2:** Quantified immunohistochemical staining intensity of collagen II, collagen X and aggrecan in chondrogenic pellets (N = 3).

	Average Staining Intensity Ratio	Chondrogenesis Day 17	Chondrogenesis Day 24
Col II	(S + HS)/S	1.58 ± 0.15*	1.40 ± 0.10*
	(KD + HS)/KD	1.59 ± 0.03**	1.30 ± 0.09*
	KD/S	0.68 ± 0.06*	0.70 ± 0.07*
	(S + HS)/(KD + HS)	0.71 ± 0.07	0.65 ± 0.04*
Col X	(S + HS)/S	1.39 ± 0.10*	1.27 ± 0.07*
	(KD + HS)/KD	1.56 ± 0.10*	1.31 ± 0.04*
	KD/S	0.64 ± 0.02*	0.77 ± 0.04*
	(S + HS)/(KD + HS	0.72 ± 0.02*	0.79 ± 0.08*
Aggrecan	(S + HS)/S	1.36 ± 0.03*	1.06 ± 0.03*
	(KD + HS)/KD	1.32 ± 0.06*	1.06 ± 0.07*
	KD/S	0.87 ± 0.11	0.87 ± 0.10
	(S + HS)/(KD + HS)	0.84 ± 0.13	0.88 ± 0.14

Chon: Chondrogenic; HS: Heat Shock; Col: Collagen; *P < 0.05; **P < 0.01.

## Discussion

Bone can regenerate itself *in vivo*, but large bone defects and slow bone growth remain obstacles. Heat shock proteins and their distribution have been observed during bone development in mouse embryos^13^. Mild periodic heat stimulation facilitated and enhanced mesenchymal stem cell differentiation toward bone and cartilage lineages^2,3^. Here, our mechanistic study revealed that HSP70 plays an essential role in osteogenesis and chondrogenesis of mesenchymal stem cells (hMSCs) as well as in thermally-induced enhancement of osteo-chondogenic differentiation. Our results demonstrated that inhibition of HSP70 with HSP70 shRNAs led to a significant reduction in the expression of osteogenic markers (e.g. ALP activity, calcium deposition, Runx2 and Osterix), chondrogenic markers (e.g. collagen II), and hypertrophic cartilage markers (e.g. type X collagen) during osteogenic and chondrogenic differentiation of hMSCs, respectively. Mild periodic heat shock to hMSCs with downregulated HSP70 cannot restore the normal osteogenic and chondrogenic differentiation.

Owing to the potential cytotoxic and off-target effects of chemical inhibitors, we used target-specific HSP70 shRNAs to knock down HSP70 expression. The data from hMSCs with scrambled shRNA indicates that osteogenic functions of hMSCs were not affected by shRNA transfection during differentiation. Furthermore, HSP70 silencing up to ~70% did not affect hMSC proliferation or hMSC surface marker expression. In contrast, studies from Jaattela *et al*. showed that high expression of HSP70 was required for the survival of glioblastoma, breast and colon carcinoma^24,25^. They showed that RNA interference-based knockdown of HSP70 resulted in massive cell death in various cancer cell lines such as MDA-MD-468, LoVo-36, U373MG, and PC-3^24^. Interestingly, Jaattela *et al*. also found that the survival of noncancerous breast epithelial cells or fetal fibroblasts was not affected by the inhibition of HSP70 in spite of the massive death of human breast cancer cells^26,27^. However, other studies reported that individual silencing of HSP70 had no significant effect on proliferation and apoptosis of HCT116 and A2780 cancer cells^28,29^. We speculate that differences in the extent of cell death and proliferation observed in the various studies involved in HSP70 depletion are due to inherent differences in the various cell types used in the respective studies. It is well known that primary healthy cells have lower growth rates than cancer cells. The fast and abnormal metabolic reactions in cancer cells may cause more protein aggregations than in healthy cells, leading to a higher demand for HSP70 molecules as chaperones in cancer cells than that in healthy cells.

To verify the specificity of HSP70 shRNA, the expression of HSP27 and HSP90 mRNA was examined in hMSCs transfected with HSP70 shRNAs and the control plasmid. The HSP70 shRNA used in this study had no effect on HSP27 and HSP90 expression in hMSCs as indicated by the lack of difference in the expression of HSP27 and HSP90 between hMSCs knocked down with HSP70 shRNA and hMSCs infected with scrambled shRNA during osteogenic differentiation on Days 12 and 26 (see Figure S1 in Supplement). In this study, heat-enhanced osteogenesis and chondrogenesis using hMSCs transfected with scrambled shRNAs and periodically heated at 39°C once every other day was first confirmed, which was consistent with our previous study using a heating protocol of 41 °C once a week in hMSCs without shRNA transfection^2,3^. Furthermore, a direct and essential role of HSP70 in MSC osteogenesis and chondrogenesis was revealed even though the expression level of HSP70 in hMSCs during osteogenic and chondrogenic differentiation is low.

A need for more chaperone proteins (e.g. HSP70) most likely is caused by quicker biochemical reactions, higher protein synthesis levels and more protein aggregations during stem cell differentiation^30,31^ than a relative equilibrium state of slow stem cell growth. However, exact signaling pathways of HSP70 interacting with MSC osteogenesis and chondrogenesis are not clear. It is widely known that bone morphogenetic proteins (BMPs), members of the TGF-β superfamily, promote osteogenic differentiation indicated by increased expression of osteoblast specific makers such as ALP, calcium deposition, Osterix, and Runx2 after BMP stimulation^32,33^. Involvement of BMPs in the induction of chondrogenesis in primary cultures of articular chondrocytes has been demonstrated *in vitro*, showing BMPs increased the expression of collagen II^34,35^ and X^35^ and aggrecan^36^. Our previous studies revealed that periodic heat shock actually enhanced BMP2 expression during the late stages of osteogenic differentiation of hMSCs in 2D culture and in 3D culture on PuraMatrix peptide hydrogels^2^. The signaling pathways associated with thermal enhancement of MSC differentiation and HSP70 involvement with MSC osteogenesis and chondrogenesis are likely more complicated than the action of BMP2 alone. For example, it was reported that BMP2 activated mitogen-activated protein kinase components, such as p38 and Erk1/2, during osteoblast differentiation, while phosphorylated p38 and Erk1/2 also mediate BMP2-induced Osterix expression^37,38^. More and in depth investigations are needed to unveil the exact signaling pathways that drive HSP70-mediated MSC osteogenesis with or without thermal enhancement.

*In vitro* chondrogenesis of hMSCs provides a useful model for studying the cellular differentiation into the chondrogenic lineage using a high cell density pellet culture system^39^. HSP70 knockdown in hMSCs by shRNAs resulted in a significant reduction of collagen II and X in heated and non-heated chondrogenic pellets during chondrogenesis. Collagen type II is a major component of hyaline cartilage and collagen X is considered a marker of hypertrophic chondrocyte differentiation in articular cartilage. Kubo and Tonomura *et al*. investigated the role of HSP70 in chondrocytes and revealed HSP70 as positive mediator in the protection of chondrocytes against extreme heat stress via inhibition of NO-induced apoptosis and promotion of metabolic activity of chondrocytes^40,41^. Supporting our findings, it was shown that HSP70 was elevated in rabbit articular chondrocytes concomitant with enhanced expression of collagen II and proteoglycan core protein after low energy microwave stimulation^42^. Direct upregulation of HSP70 via a vector in a human chondrocyte-like cell line (HCS-2/8) resulted in increased mRNA expression of proteoglycan core protein^43^. Aggrecan is the most abundant structural proteoglycan in articular cartilage and plays an important role in mediating chondrocyte-chondrocyte and chondrocyte-matrix interactions^44^. Interestingly, our data showed that HSP70 knockdown did not significantly affect aggrecan expression in chondrogenic pellets. The discrepancy might indicate that HSP70 is not the only regulatory molecule that directs aggrecan core protein expression. More investigations into how HSP70 interacts with collagen II and X but not aggrecan during MSC chondrogenesis will help elucidate the specific mechanisms of HSP70-mediated thermal enhancement of chondrogenesis.

Overall, this study revealed a direct and positive regulation of HSP70 on hMSC osteogenic and chondrogenic differentiation *in vitro*. Further work is underway to use a vector to upregulate HSP70 expression without heat shock and to examine that HSP70 upregulation alone without heat shock can enhance hMSC osteogenic and chondrogenic differentiation. There is a potential to develop new therapeutic strategies (e.g. ultrasound heating) using heat shock or heat shock proteins for accelerated bone regeneration via MSC osteogenesis and chondrogenesis to benefit a speedy recovery from trauma or pathological/physiological bone loss.

## Methods

### Antibodies and Chemicals

All reagents and chemicals were purchased from Sigma-Aldrich if they are not listed separately. HSP70 antibody was purchased from Santa Cruz Biotechnology. The following antibodies were used in flow cytometry analysis of MSC characterization, PE CD29, PE CD34, PE CD44, FITC CD45, FITC CD147, PE IgG1, and FITC IgG1 (BD Biosciences).

### Bone Marrow hMSC Isolation, Characterization, and Culture

Human MSCs were isolated from fresh human bone marrow purchased from AllCells (Alameda, CA) according to manufacturer’s instructions as previously described^2^ and cultured with MSC growth medium consisting of the Dulbecco’s modified Eagle’s medium-low glucose, 10% fetal bovine serum (Atlanta Biologicals, Lawrenceville, GA), and 1% penicillin–streptomycin (Invitrogen, Carlsbad, CA) in an incubator at 37 °C with 5% CO_2_ at a density of 5,000 cells/cm^2^. Human MSCs at Passage 4 with and without HSP70 knockdown were characterized using cell surface markers by a FACSCalibur flow cytometer (BD Biosciences) as previously described^2^. IgG1 immunoglobulin was used as a negative control. In this study P4 hMSCs were used in all experiments, which were run in triplicate and repeated three times.

### HSP70 Knockdown in hMSCs by shRNAs

HSP70 expression was knocked down by shRNA mediated gene silencing using the lentiviral technique. Six shRNAs were tested targeting human HSPA1A and HSPA1B from Sigma Aldrich in the pLKO.1 lentivirus vector. A scrambled shRNA was used as a control. HEK293T cells were kindly provided by Dr. Xuejun Jiang’s lab at Memorial Sloan Kettering Cancer Center as a part of the lentivirus packaging system. Lentivirus was produced by co-transfecting HSP70 HSPA1A/HSPA1B shRNA pLKO.1 vector into HEK293T cells using Lipofectamine (Invitrogen, Carlsbad, CA) according to the manufacturer’s instructions. Human MSCs at Passage 3 were exposed to 39 °C for one hour followed by 16 hours incubation at 37 °C to enable a high level of HSP70 expression^45^ before transfection in the presence of polybrene (3.4 µg/ml) and selected with puromycin (2 µg/ml). The individual puromycin-resistant clones were chosen for further studies.

### Confirmation of Gene Silencing by Western Blotting and Reverse Transcription PCR

Human MSCs transfected with HSP70 shRNAs were harvested 14–16 hours after mild heating at 39 °C for one hour. Protein gel blotting analyses were performed as previously described^2^. Briefly, cells were lysed in lysis buffer (150 mM NaCl, 1% NP-40, 0.5% Deoxycholic Acid, 0.1% SDS, 50 mM Tris pH 8.0) containing protease inhibitor cocktails (Sigma, St. Louis, MO). Proteins were separated with SDS-polyacrylamide gel electrophoresis, transferred to PVDF membrane (Bio-Rad, Hercules, CA) and immunoblotted with the primary antibodies of HSP70 and actin, followed by incubation with the horseradish peroxidase (HRP) conjugated secondary antibody. Tetramethylbenzidine (TMB) substrate kit (Vector Laboratories, Burlingame, CA) was used to visualize the protein bands. Membranes were dried and scanned into digital images. Protein bands were analyzed quantitatively using Image Pro Plus software (Media Cybernetics). Protein expression levels were presented as ratios of total intensity of the protein bands normalized by that of the actin band to minimize the protein loading variation.

For RT-PCR, briefly, total RNA from hMSCs pellets was isolated using RNeasy Kit (Qiagen) following reverse transcription by SuperScript III RT (Invitrogen). TagDNA polymerase (Invitrogen) was used in PCR cycles and β-actin was used as a control. Primers for human HSP70 and β-actin are CCATGGTGCTGACCAAGATGAAG (forward)/CACCAGCGTCAATGGAGAGAACC (reverse), and CCAGAGCAAGAGAGGCATCC (forward)/CTGTGGTGGTGAAGCTGTAG (reverse) respectively^46^.

### Human MSC Osteogenic Differentiation

Ten thousand (10^4^) cells at passage 4 were seeded per well in 24-well plates. Osteogenic differentiation was induced the next day (Day 0) after seeding using osteogenic medium consisting of growth medium with 50 µM ascorbic acid phosphate (Wako Chemicals USA, Richmond, VA), 10 mM β-glycerophosphate, and 0.1 µM dexamethasone.

### Human MSC Chondrogenic Differentiation

Pellet culture was performed for chondrogenic differentiation of hMSCs as previously described^3^. Briefly, 2.5 × 10^5^ hMSCs at passage 4 in 0.5 ml medium were centrifuged down in a 15 ml conical tube at 150Xg for 5 minutes. Pellets were cultured in an incubator at 37 °C with 5% CO_2_ and humidified air. Chondrogenic differentiation was induced by chondrogenic medium composed of DMEM-high glucose, supplemented with 1% ITS + Premix (BD Bioscience), 1% penicillin-streptomycin, 100 μg/ml sodium pyruvate (Invitrogen), 50 μg/ml ascorbic acid-2-phosphate, 40 μg/ml L-proline, 0.1 μM dexamethasone, and 10 ng/ml recombinant human transforming growth factor- β3 (TGF-β3) (Lonza, Walkersville, MD).

### Heat Exposure

Human MSCs were exposed to mild heating at 39 °C for one hour once every other day starting Day 1 in an incubator with 5% CO_2_ and humidified air. After heating, the medium was changed to avoid any possible loss of water, and the cells were returned to the 37 °C incubator. The medium change was also performed for the non-heated culture samples.

### dsDNA Quantification

dsDNA content was determined on Days 6, 12, 19, and 26 during differentiation using Quant-iT^TM^ PicoGreen dsDNA reagent kit (Invitrogen) following the manufacturer’s instructions. Briefly, 0.5% Triton-X 100 was used to lyse cells to release DNA. The cell lysate was incubated with pepsin overnight at 4 °C. Samples were neutralized with pH 8.0 Tris buffer, and incubated with PicoGreen working solution for 5 minutes at room temperature. The fluorescence signals were measured using a SpectraMax M2e microplate reader (Molecular Devices) at excitation and emission wavelengths of 480 nm and 520 nm, respectively.

### Alkaline Phosphatase Assay

ALP activity was measured on Days 6 and 12 during differentiation as previously described^2^. Briefly, cells were lysed with 0.5% Triton-X 100. Samples were incubated with 1.5 M alkaline buffer solution and phosphatase substrate solution for 15 minutes at 37 °C. After incubation, 1 N NaOH was added to stop the reaction. The absorbance of the p-nitrophenol product was measured at 405 nm using the SpectraMax M2e microplate reader. One enzyme unit of ALP is defined as the quantity of enzyme which produces 1nmol p-nitrophenol per 15 minutes.

### Calcium Quantification

Calcium deposition was performed on Days 19 and 26 during differentiation as previously described^2^. Briefly, samples were collected using 0.5 N HCl and calcium was extracted by shaking the samples for 4 hours at 4 °C. Sample calcium concentrations were measured using the Stanbio Laboratory Calcium Liquicolor kit (Fisher Scientific).

### Gene Expression Measured by Real-time Quantitative RT-PCR

Real-time RT-PCR was performed for HSP70, Runx2, Osterix, and GAPDH using SYBR Green RT-PCR Master Mix (Life Technologies). Real-time quantitative RT-PCR was performed using the ABI Prism 7300 Thermocycler (Applied Biosystems) following the manufacturer’s instructions. The thermal profile for real-time PCR was 95 °C for 10 min, followed by 40 cycles of 95 °C for 15 s and 60 °C for 1 min. Relative gene expression levels from real-time PCR experiments were assessed using the 2^−∆∆CT^ method^47^. Primers for human Runx2, Osterix, and GAPDH are AAATCGCCAGGCTTCATA (forward)/CTGCCAGGAGTGGTCAAA (reverse); CCTGCGACTGCCCTAATT (forward)/GCGAAGCCTTGCCATACA (reverse); and GGATTTGGTCGTATTGGG (forward)/GGAAGATGGTGATGGGATT (reverse) respectively^2^.

### Histology and Immunohistochemistry (IHC)

Chondrogenic pellets were harvested for histological analyses on Days 17 and 24 during differentiation. Immunohistochemistry for HSP70, collagen type II, type X, and aggrecan was performed on chondrogenic pellets as previously described^3^.

### Statistical analysis

Data are reported as mean ± standard deviation with a sample number of four for each condition. Student’s t-tests were applied to analyze surface marker expression from FACS and dsDNA data for proliferation of hMSCs. ANOVA with *post hoc* Holm-Sidak tests were performed for all the other experiment data. A *P* value of less than 0.05 was considered as statistically significant.

### Data Availability

The datasets generated and/or analyzed during the current study are available from the corresponding author on request.

## Electronic supplementary material

Supplementary materials
