# Supplementary material for: Downregulation of Heat Shock Protein 70 Impairs Osteogenic and Chondrogenic Differentiation in Human Mesenchymal Stem Cells
Source: Sci Rep. 2018 Jan 11;8:553. doi: 10.1038/s41598-017-18541-1 (PMC5765044; doi:10.1038/s41598-017-18541-1)
Supplement: Supplementary file 1 — Supplementary materials [file 41598_2017_18541_MOESM1_ESM.pdf]

Supplementary Materials

Downregulation of Heat Shock Protein 70 Impairs Osteogenic and Chondrogenic Differentiation in Human Mesenchymal Stem Cells

Chenghai Li, Kristifor Sunderic, Steven B. Nicoll and Sihong Wang*

***Corresponding author**:

Sihong Wang, Ph.D.
Biomedical Engineering Department, Steinman Hall 434
City College of New York
160 Convent Avenue
New York, NY 10031
USA

Phone: 212-650-5189
Fax: 212-650-6727
Email: shwang@ccny.cuny.edu

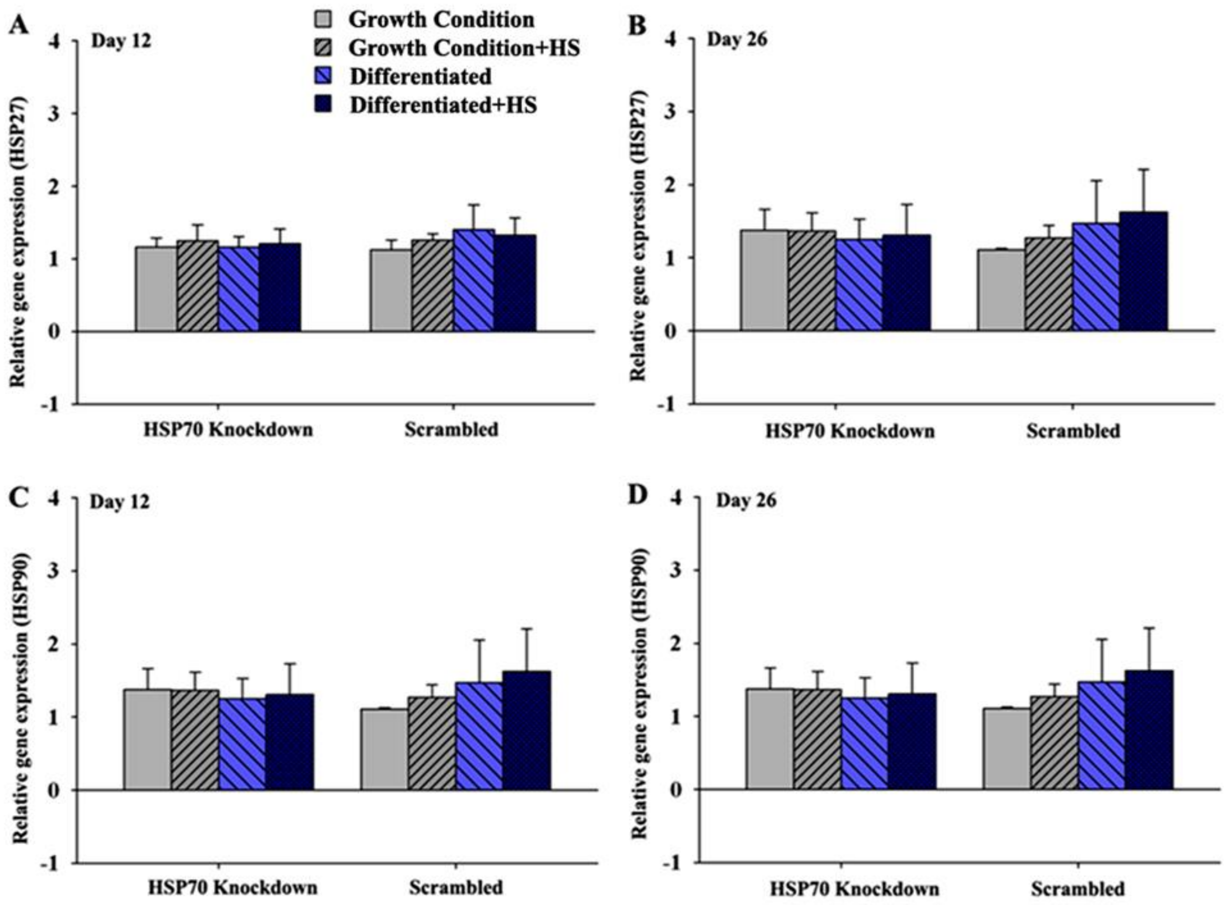

Figure S1. HSP27 and HSP90 expression measured by Real-time RT-PCR in hMSCs during the growth and differentiation at Day 12 (A and C) and Day 26 (B and D).

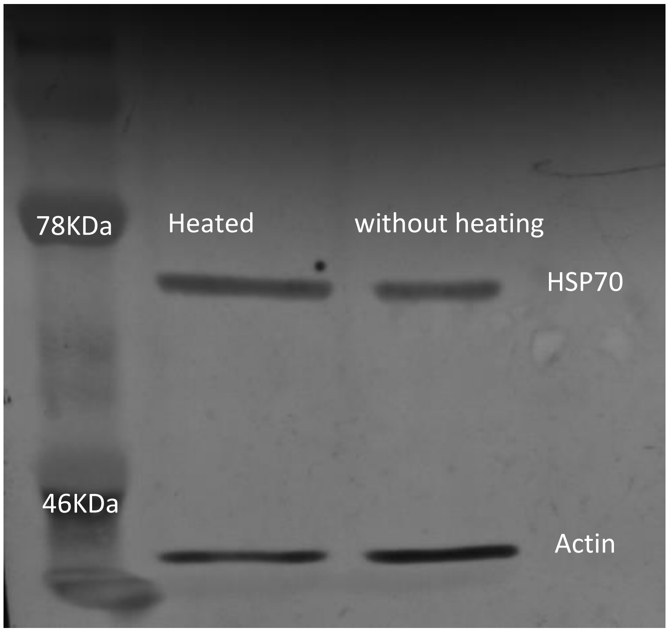

Figure S2. Western Blot membrane of Figure 1A.

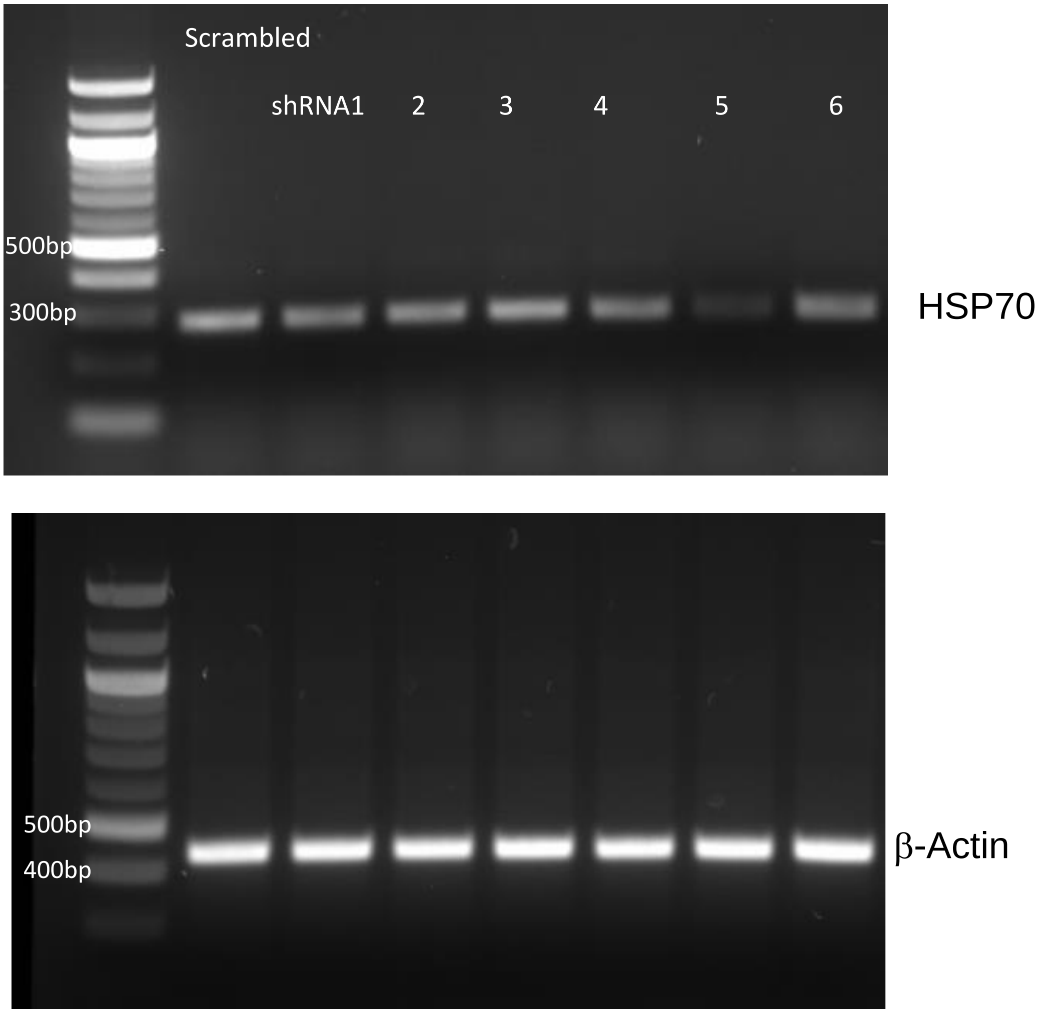

Figure S3. Full DNA gel images of Figure 2A.

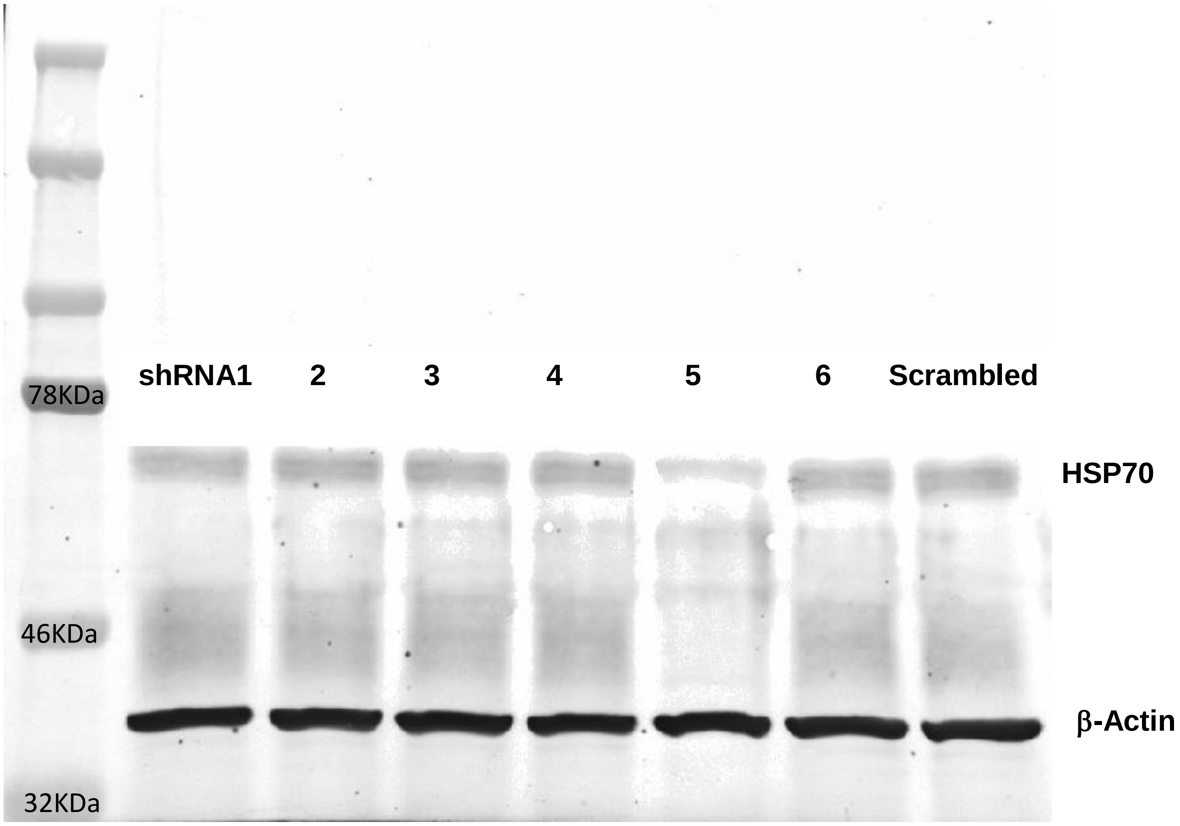

Figure S4. Full Western Blot membrane of Figure 2B.

Table S1. Alkaline Phosphatase Activity (Unit†)

		Scrambled		HSP70 Knockdown		Reduction (%)
**Day 6**						
Growth Condition		54.44±3.37		39.40±9.20		
Growth Condition+HS	*	58.26±0.36		43.92±8.42	P=0.97	
Differentiated		99.54±19.18	**	39.14±2.25		60.70
Differentiated+HS	*	156.76±20.32	**	45.30±0.87	*	71.07
**Day 12**						
Growth Condition		76.42±11.82		59.53±2.30		
Growth Condition+HS	P=0.06	85.46±12.80		70.65±4.55	*	
Differentiated		104.30±14.61	P=0.14	84.78±11.08		18.72
Differentiated+HS	*	149.09±14.96	*	107.45±14.61	P=0.09	27.93

†One enzyme unit of ALP is the amount of enzyme which releases 1 nmol p-nitrophenol per 15 minutes. Each sample had 10^4 seeded hMSCs. HS: heat shock;*P<0.05; **P<0.01. N=4.

Table S2. Calcium Deposition (μg/10^4 seeded hMSCs)

		Scrambled		HSP70 Knockdown		Reduction (%)
**Day 19**						
Growth Condition		20.94±7.10		14.66±4.12		
Growth Condition+HS	**	16.56±5.37		16.24±7.23	*	
Differentiated		173.78±13.03	**	43.88±12.51		74.75
Differentiated+HS	*	202.26±6.87	**	83.11±10.96	*	58.91
**Day 26**						
Growth Condition		61.73±3.60		51.14±6.95		
Growth Condition+HS	**	70.48±10.22		55.58±7.52	**	
Differentiated		275.57±5.65	**	160.29±10.45		41.84
Differentiated+HS	*	306.43±11.10	**	186.37±12.17	*	39.18

**P*<0.05; ***P*<0.01. N=4.
